# Plasma proteome alterations by MAPK inhibitors in *BRAF^V600^*-mutated metastatic cutaneous melanoma^☆^^☆☆^

**DOI:** 10.1016/j.neo.2021.06.002

**Published:** 2021-07-08

**Authors:** Haris Babačić, Hanna Eriksson, Maria Pernemalm

**Affiliations:** a Department of Oncology-Pathology, Science for Life Laboratory, Karolinska Institute, Stockholm, Sweden; b Theme Cancer / Department of Oncology, Karolinska University Hospital, Stockholm, Sweden

**Keywords:** Melanoma, Plasma proteins, Mitogen-activated protein kinase, BRAF and MEK inhibitors, Targeted therapy, Biomarkers, V600E mutation

## Abstract

Approximately half of metastatic cutaneous melanomas (CM) harbor a mutation in the *BRAF* protooncogene, upregulating the mitogen-activated protein kinase (MAPK)-pathway. The development of inhibitors targeting the MAPK pathway (MAPKi), i.e., BRAF- and MEK-inhibitors (BRAFi and MEKi), have substantially improved the survival in *BRAF^V600E/K^*-mutated stage IV metastatic CM. However, most patients develop resistance to treatment and no predictive biomarkers exist in practice. This study aimed at discovering plasma proteome changes during treatment MAPKi in patients with metastatic (stage IV) CM. Matched plasma samples before (pre) and during treatment (trm) from 23 patients with stage IV CM, treated with BRAF-inhibitors (BRAFi) alone or BRAF- and MEK- inhibitors combined (BRAFi and MEKi), were collected and analyzed with targeted proteomics by proximity extension assays. Additionally, plasma from 9 patients treated with BRAFi and MEKi was analyzed with in-depth high-resolution isoelectric focusing liquid-chromatography mass-spectrometry proteomics. Alterations of plasma proteins involved in granzyme and interferon gamma pathways were detected in patients treated with BRAFi, and cell adhesion-, neutrophil degranulation-, and proteolysis pathways in patients treated with BRAFi and MEKi. Several proteins were associated with progression-free survival after MAPKi treatment. We show that the majority of the altered plasma proteins were traceable to *BRAF^V600E^*-mutant metastatic CM tissue at mRNA level in 154 patients from the TCGA, further strengthening their involvement in tumoral response to treatment. This wide screen of plasma proteins unravels proteins that may serve as predictive and/or prognostic biomarkers of MAPKi treatment, opening a window of opportunity for plasma biomarker discovery in MAPKi-treatment of *BRAF^V600^*-mutant metastatic CM.

## Highlights

• Based on antibody-based proteomics by proximity extension assays, pre-treatment levels of several proteins were predictive of shorter progression-free survival (PFS) after treatment with MAPK-inhibitors in metastatic cutaneous melanoma (CM), including IL6, IL10, CCL-2/-3/-4, LGALS1, and CSF1.
• By in-depth mass-spectrometry-based proteomic analysis of 1,160 proteins in a subset of metastatic CM patients receiving BRAF- and MEK- inhibitors, we discovered alterations in plasma proteins involved in cell adhesion-, neutrophil degranulation-, and proteolysis- during BRAFi and MEKi treatment.
• CPB1 had the highest increase during BRAF- and MEK- inhibitors’ treatment and was associated with longer PFS.
• Most of the proteins altered in plasma during MAPKi treatment were traceable to *BRAF^V600E^*-mutated metastatic CM tissue at mRNA level, based on expression patterns in 154 patients from the TCGA cohort.

## Introduction

Approximately 50% of cutaneous melanomas (CM) harbor an activating mutation in the B-Raf protooncogene (*BRAF*), predominantly a substitution of valine to glutamine in codon 600 (i.e., *BRAF^V600E^*), conferring a constitutive activation of the mitogen-activated protein kinase (MAPK)-pathway, which stimulates tumor cell proliferation and survival [1]. Combination therapy with BRAF- and MEK- inhibitors (BRAFi and MEKi) induces rapid and more effective therapy responses than BRAFi monotherapy in *BRAF^V600E/K^* disease [2], [3], [4]. Combination therapy with dabrafenib and trametinib showed progression-free survival (PFS) of 21% at 4 years and 19% at 5 years [2]. The corresponding overall survival (OS) rates were 37% and 34% at 4 and 5 years, respectively. In comparison, treatment with immune checkpoint blockade (ICB) induces durable clinical responses in over 45% of the patients with *BRAF*-mutated melanoma [5]. However, a subgroup of patients with BRAF wild-type and BRAF*^V600^*-mutant CM seem to have comparable rates of 4-year PFS and OS, respectively, with a small but significant difference of 40% as compared to 34% objective response rates according to mutation status [6]. Two retrospective analyses have suggested that *BRAF^V600^*-mutant CM patients have a better outcome after a frontline treatment with ICB [7,8].

Presently, *BRAF^V600^* mutation status is the only validated biomarker for MAPKi treatment in metastatic CM. Usually resistance to targeted therapy occurs within 6 to 12 months, but a subset of patients receiving BRAFi and MEKi reaches long-term responses, which have been correlated to normal lactate dehydrogenase (LDH) levels and few metastatic sites [2,9,10]. Validated biomarkers for therapy selection are crucial in improving treatment decisions, but are still lacking for metastatic CM.

Protein-based liquid biopsy approaches in blood indicative of response to treatment would be extremely useful, as they are easy to sample, quick, cheap, and allow for longitudinal sampling. However, due to the complexity of the plasma proteome, which has a wide range of protein concentrations, proteomic methods have been limited in discovering new associations between plasma proteins and response to MAPKi treatment in metastatic CM. We have previously pushed the limits in investigating the plasma proteome by unbiased mass-spectrometry-based in-depth proteomics with high resolution isoelectric focusing, liquid chromatography, and mass spectrometry (HiRIEF LC-MS/MS) [11], allowing for relative quantification of over 1,000 proteins in plasma, including circulating and tissue-derived proteins [12]. Recently, we have demonstrated that combining HiRIEF LC-MS/MS with targeted proteomic analyses provides a unique and previously unexplored view into the dynamic changes of the proteins present in plasma of metastatic melanoma patients receiving anti-PD-1 ICB, and the ability to detect protein-coding sequence variants in plasma [13].

The aim of this explorative study was to analyze the plasma proteome changes in patients with metastatic (stage IV) CM during treatment with first-line MAPKi, using both antibody-based, targeted proteomics with proximity extension assays (PEA) and unbiased global proteomics with HiRIEF LC-MS/MS [11,14], to discover potential biomarkers predicting duration of treatment response. However, one challenge in plasma proteomic biomarker discovery lies in identifying where do the altered plasma proteins come from. Therefore, to further explore whether the candidate plasma protein biomarkers originate from metastatic CM tissue, through a bioinformatic approach using TCGA data, we further demonstrate that most of the proteins deregulated in plasma during MAPKi-treatment are traceable to *BRAF^V600E^*-mutated metastatic CM tissue at mRNA level.

## Materials and methods

Details on patients and methods are provided in Supplementary File 1 and [13]. Brief description is provided below.

### Ethics statement

All patients gave informed consent for the study. The study was performed according to the guidelines of Good Clinical Practice and the Declaration of Helsinki, and approved by the Stockholm Regional Ethics Committee, Karolinska Institute, Sweden, application number: 2011/1980-31/1.

### Patients and plasma samples

Forty-eight serial blood samples collected before (pre-trm) and after the first treatment cycle (trm) from 24 metastatic CM patients treated with first-line MAPKi (BRAFi alone or BRAFi and MEKi in combination) were collected. The plasma samples were centrifuged of the blood containing EDTA anticoagulant and stored at -70°C until proteomic analyses.

The patients were followed longitudinally and clinical data was collected for further analyses: age at treatment start, sex, baseline M-stage according to AJCC8 [15], baseline LDH levels, best response to treatment, date of disease progression and date of death. Best response was defined according to clinical and radiological investigations as complete response (CR), stable disease (SD), partial response (PR), and progressive disease (PD). PFS was defined from treatment start until date of confirmed progression or date of death of any cause, and OS as the time from treatment start until date of death of any cause.

### Mass-spectrometry-based proteomics

The mass-spectrometry plasma proteomics method has previously been described in detail [12]. In brief, due to the complexity of the plasma proteome, with a wide range of protein concentrations, we depleted the high abundant proteins with Agilent Plasma 14 Multiple Removal System 4.6 × 100 (Agilent Technologies). After depletion, the plasma was denatured at 95°C for 5 minutes, and proteins reduced with dithiothreitol and alkylated with iodoacetamide. After digestion with LysC (1:50 ratio) at 37°C overnight, the samples were additionally digested by trypsin (1:50 ratio) at 37°C overnight. Equal amounts of peptide solutions in the samples were pH adjusted with TEAB, pH 8.5, and then labelled with tandem mass tags (TMT)-10. We distributed pre-trm and trm samples equally across 5 TMT10 sets, in which we have also equally distributed samples of patients treated with anti-PD-1 ICB [13].

After labeling, the peptide samples were fractionated using the HiRIEF prefractionation method, described elsewhere [11]. Subsequently, the fractions were dried in the freezer and kept at -20°C until LC-MS/MS analysis.

### Proximity extension assays

The plasma samples were analyzed by proximity extension assays (PEA), a targeted, antibody-based, proteomics method at the Clinical biomarkers’ facility at SciLifeLab, Uppsala, Sweden. We used the PEA Olink ImmunoOnc I panel, which targets 92 immuno-oncology biomarkers (Table S0 – Supplementary File 2). In brief, 2 oligonucleotide-labeled antibodies bind to a targeted protein, followed by hybridization of the oligonucleotides if the probes are in proximity. Adding DNA polymerase to the sample generates a unique PCR target sequence, which is then detected and quantified with a microfluidic real-time PCR instrument (Biomark HD, Fluidigm). The data are then quality controlled, normalized with an internal standard, and then presented with normalized protein expression values (NPX), on a log2 scale. Details are available at [14].

## Statistical and bioinformatic analysis

### Plasma proteomics

The proteins were annotated with gene IDs. We included only proteins with at least 80% and 50% of observations in PEA and HiRIEF data, respectively. The protein quantification levels were log2-normalised for both methods, and HiRIEF and PEA analyses were performed separately. The change in protein plasma levels was estimated with the log2 fold change (log2-FC).

Using a 2-sided paired *t* test at *α* = 0.05, we compared the trm protein plasma levels to the matching pre-trm protein plasma levels, to detect plasma protein alterations due to treatment with MAPKi. We stratified the differential analysis based on treatment type to a group treated only with BRAFi and a group treated with combination therapy of BRAFi and MEKi. We corrected for multiple testing with the false discovery rate (FDR), and interpreted the findings based on FDR in the PEA analyses, but avoided doing so in the HiRIEF LC-MS/MS analyses to avoid type II error.

Associations between protein plasma levels and PFS were determined with Cox proportional hazards models, based on a likelihood ratio test (LRT) at *α* = 0.05, and corrected for multiple testing with FDR. In PEA data, where a clinical variable was associated with PFS, the associations between protein levels and PFS were adjusted for that variable. Furthermore, regardless of their association with PFS, we further adjusted in the models for age, sex and abnormal LDH levels in a sensitivity analysis. Due to the small sample size of BRAFi and MEKi subgroup, we avoided including clinical variables in the Cox models on HiRIEF data. All analyses were performed in R, v4.0.3.

### The Cancer Genome Atlas analyses

Due to the lack of an in-depth proteomics dataset of metastatic CM tissue with corresponding clinical data, we used The Cancer Genome Atlas (TCGA) data for orthogonal analyses of protein-coding gene expression in metastatic CM tissue.

Of the 467 patients from the TCGA, 308 (66%) had a mutation in the *BRAF* gene, of which 206 (44%) had a V600E mutation and none had a V600K mutation. For this study, we selected the metastatic CM samples with *BRAF^V600E^* mutation (*n* = 154) and estimated the mean log2-CPM expression in this group of patients. To categorize gene expression to high versus low in *BRAF^V600E^* metastatic CM, we calculated *z* score on mean log2-CPM expression per gene transcript. We then compared which of the proteins altered in plasma during MAPKi treatment were traceable to metastatic CM at mRNA level and whether the gene had on average higher (*z* > 0) or lower (*z* < 0) expression relative to the rest of the genes.

Finally, all log2-CPM were converted to gene expression *z* scores per patient with *BRAF^V600E^*-mutated metastatic CM, based on the mean and SD across patients, to make gene expression comparable between samples. The association between mRNA expression (*z* scores) and OS in patients with *BRAF^V600E^*-mutated metastatic CM (n = 154) was analyzed with Cox proportional hazards models, after adjusting for patient's age and sex. The association was tested with a likelihood ratio test, at *α* = 0.05, and corrected for multiple testing with FDR. The association with OS was further confirmed categorically with Kaplan-Meier curves and log-rank test.

## Results

### Patients’ characteristics

Proteomic studies have demonstrated that the inter-individual variability in the plasma proteome are overall larger than the intra-individual variability [12,[16], [17], [18], [19]]. Comparing different individuals only before or only during treatment is likely to confound plasma proteomic analyses due to individual pre-existing differences in their plasma proteomes. Consequently, analyses of matched plasma samples, collected pre-trm and during trm from the same individual, are more likely to detect biological processes stirred by the treatment.

Matched plasma samples were collected from 24 patients treated with either BRAFi alone (*n =* 15) or a combination of BRAFi and MEKi (*n* = 9). Clinical characteristics stratified on response, PFS, and OS are described in Table 1 and Figure S1, respectively. Median number of days between collecting pre-trm and trm plasma samples was 27.5 days (SD: 12.43). One patient treated with BRAFi was excluded from PEA analyses because the trm sample was of poor quality.

**Table 1:** Clinical characteristics of patients with metastatic cutaneous melanoma receiving MAPK-inhibitors, stratified according to best response.

Clinical characteristics	MAPKi-R (*n* = 16)	MAPKi-NR (*n =* 7)	*P*
Median age at treatment start (years)	51 ± 10.21	60 ± 14.73	0.389
Females – *n* (%)	7 (43.75)	0	0.019
Males – *n* (%)	9 (56.25)	7 (100.00)	
Baseline M-stage – *n* (%)			
M1a	2 (12.50)	0	1
M1b	0	0	
M1c-d	14 (87.50)	7 (100.00)	
Baseline LDH			
Median (μkat/L)	4.90 ± 4.41	5.00 ± 16.55	0.267
≦ ULN – *n* (%)	3 (18.75)	2 (28.57)	0.621
> ULN – *n* (%)	13 (81.25)	5 (71.43)	
First-line therapy – *n* (%)			
Anti-BRAF	9 (56.25)	5 (71.43)	
anti-BRAF and anti-MEK	7 (43.75)	2 (28.57)	0.657
Best treatment response – *n* (%)			
Complete response	1 (6.25)	0	
Partial response	13 (81.25)	0	NA
Stable disease	2 (12.50)	0	
Progressive disease (no response)	0	7 (100.00)	
Progression-free survival – median (days)	220.5 ± 159.97	81 ± 24.34	<0.001
Overall survival – median (days)	356 ± 420.61	182 ± 117.89	0.017

MAPKi-NR = patients treated with MAPK inhibitors with no response to treatment; MAPKi-R = patients treated with MAPK inhibitors with complete or partial response of stable disease after treatment; *P* = *P* value obtained with *t, Fisher*, or *log-rank* test, number after ± = standard deviation.

### Plasma proteomics

To detect changes in the plasma proteome related to response to treatment, we used antibody-based PEA assays in matched plasma samples from 23 patients, in order to quantify 92 low-abundant plasma proteins involved in immune processes in cancer. Proteins with more than 20% missing values were excluded from statistical analyses (*n =* 15).

**Fig. 1:**
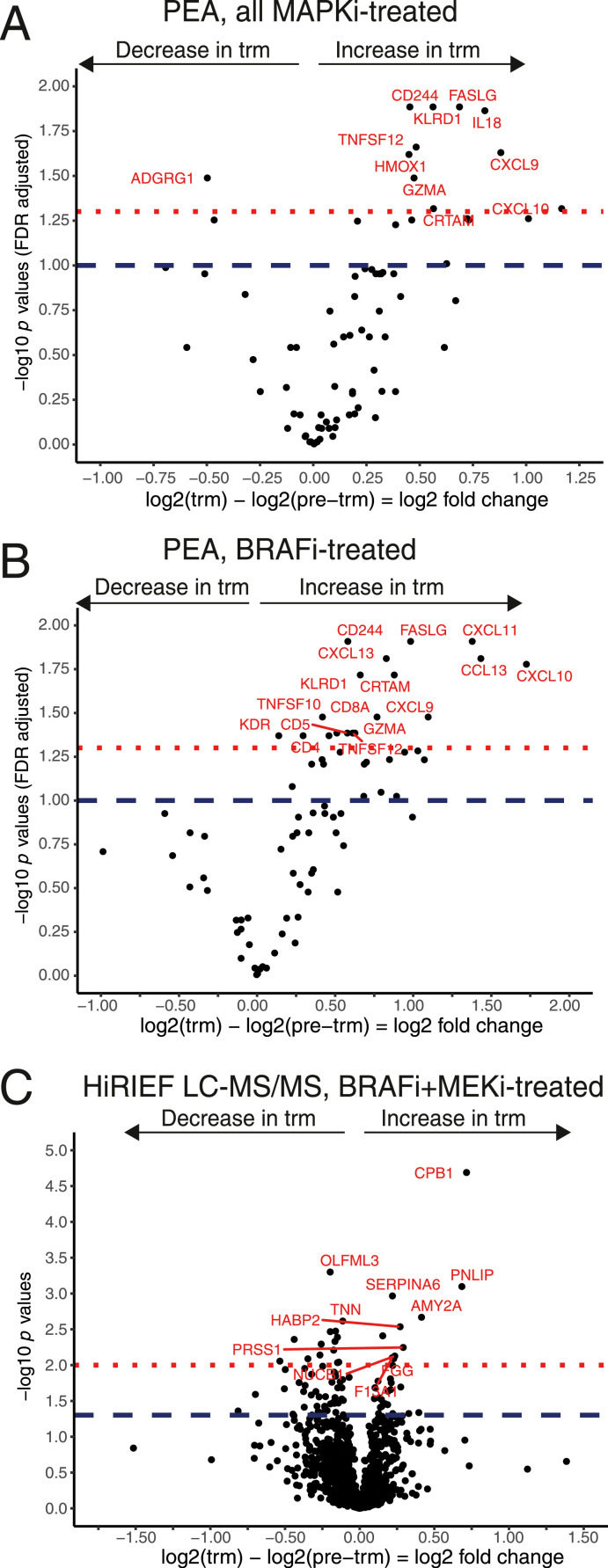
**Mean log2-fold change (log2-FC) in protein plasma levels during treatment with MAPKi:**(A) PEA data, all MAPKi-treated patients analyzed together with a paired t test, proteins above the blue dashed line = FDR-adjusted *P < 0*.1, proteins above the red dotted line = FDR-adjusted *P < 0*.05; (B) PEA data, subgroup of BRAFi-treated patients analyzed with a paired t test, proteins above the blue dashed line = FDR-adjusted *P < 0*.1, proteins above the red dotted line = FDR-adjusted *P < 0*.05; (C) HiRIEF LC-MS/MS data, subgroup of BRAFi and MEKi-treated patients analyzed with a paired t test, proteins above the blue dashed line = *P < 0*.05, proteins above the red dotted line = *P < 0*.01, no FDR adjusted.

To analyze the overall effect of MAPKi (BRAFi alone or BRAFi and MEKi in combination) on the plasma proteome, we performed a paired analysis of pre-trm and trm plasma samples. Based on PEA data, we detected a change in plasma levels of 19 proteins (t test, *P*< 0.05, 10% FDR, Figure 1A; Table S1 - Supplementary File 2), with the chemokine (C-X-C motif) ligands 10, 11, and 9 (CXCL10, CXCL11, CXCL9) and interleukin (IL18) having the largest increase, and only MICA/MICB and ADGRG1 having a decrease in plasma levels. Comparing patient responders who had disease control (CR, PR or SD) to patients who had no response to MAPKi treatment (PD) showed a non-significant trend (> 10% FDR) of higher pre-treatment plasma protein levels of 12 proteins (Table S2 – Supplementary File 2). Comparing the log2-FC between responders and non-responders to MAPKi treatment showed an inverse state, where responders seemed to have had a larger decrease in CXCL1, CXCL5 and ADGRG1, but the findings were also not significant (Table S3 – Supplementary File 2).

### BRAFi-associated plasma proteome alterations

To explore whether the 2 treatments have driven similar or different processes, we stratified the MAPKi cohort based on treatment type, to BRAFi treatment (*n =* 15) and BRAFi and MEKi treatment (*n =* 9). According to PEA, a total of 32 proteins were differentially altered in the BRAFi subgroup during treatment (t test, *P < 0*.05, 10% FDR, Figure 1B; Table S4 - Supplementary File 2). Worth noticing is that all the proteins that had an increase in the entire MAPKi cohort also had an increase in the BRAFi subgroup as well. Surprisingly, we detected an increase in programmed cell-death protein 1 (PD-1) levels during treatment in the BRAFi subgroup (*n =* 14) when analyzed separately (log2-FC=0.70, p=0.0196, FDR q=0.060), which was not previously observed in the full MAPKi cohort [13]. An association between pre-trm PD-1 plasma levels and PFS and OS after anti-PD-1 ICB treatment has been recently reported, but the authors found no association between pre-trm PD-1 plasma levels and PFS or OS in patients treated with BRAFi [20]. BRAFi-treated responders had a non-significant trend of higher pre-treatment plasma levels of MMP12, CCL19, and CXCL1 and lower HMOX1 levels, compared to BRAFi non-responders, and had no difference in log2-FC in plasma proteins during treatment.

### BRAFi and MEKi-associated plasma proteome alterations

Since BRAFi and MEKi combination treatment shows a better treatment effect than BRAFi alone [2] and is the current standard of treatment, we performed in-depth proteomic analyses combining PEA and HiRIEF LC-MS/MS, to expand the coverage of the plasma proteome in this subgroup. In total, 1,160 proteins were detected in at least 50% of the samples and were included in the statistical analyses.

Only 6 proteins appeared differentially altered during BRAFi and MEKi treatment, based on PEA data, but none of them were significant at 10% FDR. Among these, IL18 was the only one with an increase, whereas the remaining proteins decreased during treatment: TNFRSF12A, MICA/MICB, MMP12, ADGRG1, and IL10. We did not compare responders to non-responders in the BRAFi and MEKi-treated group, because there were only 2 patients with no response to treatment.

The HiRIEF LC-MS/MS analysis detected alterations in plasma levels of 84 out of 1,160 proteins during BRAFi and MEKi treatment (t test, *P < 0*.05, no FDR, Figure 1C; Table S5 - Supplementary File 2). Proteins with the largest increase were carboxypeptidase B1 (CPB1), pancreatic lipase (PNLIP), amylase alpha 2A (AMY2A), FTL, and CECR1, with CPB1 being the only protein to remain significant at 5% FDR (p = 0.00002, q = 0.0237). The most prominent decrease during treatment was observed in plasma levels of CHL1, LMNA, CHI3L1, LIPG, PTPRZ1, NPPA, and PLS3. Half of these proteins were involved in either cell adhesion, proteolysis, neutrophil degranulation or multicellular organism development (Figure S2) [13]. This highlights that global analyses with HiRIEF LC-MS/MS allow for detection of a large number of non-cytokine plasma proteins in an unbiased manner, thus finding associations initially not hypothesized about.

### Protein plasma levels and PFS

In order to determine whether the plasma proteins could be useful in predicting duration of treatment response, we analyzed the association between pre-trm plasma levels and PFS.

**Fig. 2:**
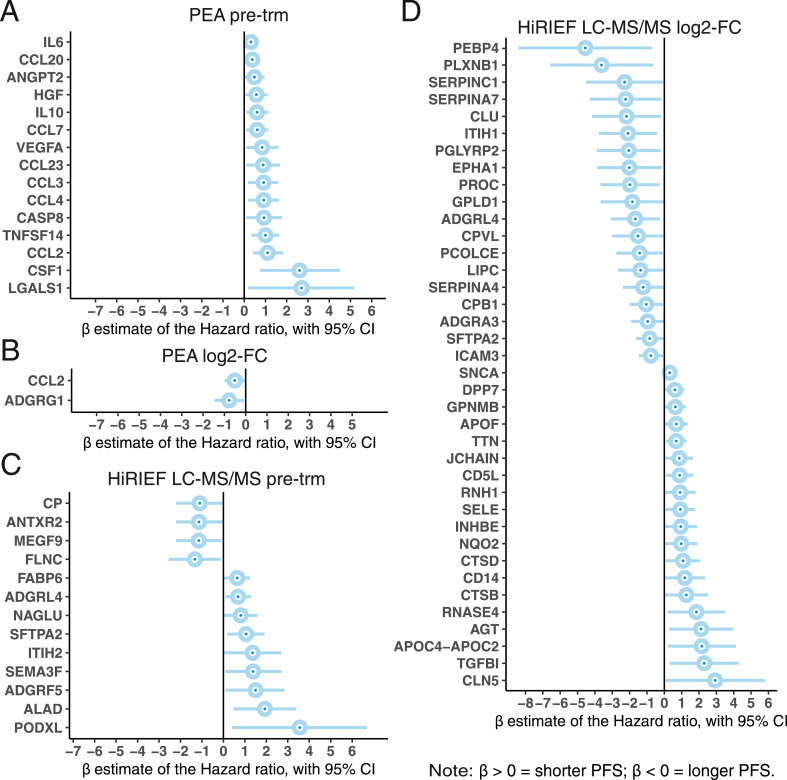
**Proteins with plasma levels associated with PFS: (**A) pre-trm levels in the full MAPKi cohort (*n =* 23), PEA data, adjusted for response to treatment (yes/no), likelihood ratio test, *P < 0*.05, 10% FDR; (B) log2-FC levels in the full MAPKi cohort (*n =* 23), PEA data, adjusted for response to treatment (yes/no), likelihood ratio test, *P < 0*.05, 10% FDR; (C) pre-trm levels in the BRAFi and MEKi subgroup (*n =* 9), HiRIEF LC-MS/MS data, univariate Cox models, likelihood ratio test, *P < 0*.05, no FDR; (D) log2-FC levels in the BRAFi and MEKi subgroup (*n =* 9), HiRIEF LC-MS/MS data, univariate Cox models, likelihood ratio test, *P < 0*.05, no FDR.

In the full cohort analyzed with PEA (*n =* 23), therapy response was the only clinical variable associated with PFS. After adjusting for it, pre-trm levels of 15 proteins were associated with shorter PFS (p_coefficient_ < 0.05, LRT *P < 0*.05, 10% FDR, Figure 2A). We have previously reported that 11 of these proteins were associated with PFS in anti-PD-1 ICB treatment as well: CCL2, CCL3, CCL4, VEGFA, ANGPT2, CSF1, HGF, IL10, IL6, LGALS1, and TNFSF14 [13], suggesting that higher pre-trm plasma levels of the majority of these proteins were associated with shorter PFS, regardless of treatment. These proteins are likely generic plasma biomarkers for poor prognosis in advanced melanoma.

However, 4 additional proteins were specifically associated with shorter PFS in the MAPKi cohort: CCL7, CCL20, CCL23, and CASP8. Because age, sex, and abnormal LDH levels (above vs. below limit of normal) could have confounded the association between protein levels and PFS [9,10], we have arbitrarily adjusted for them in a sensitivity analysis, although these clinical variables were not univariately associated with PFS in our study group. Most of the proteins remained associated with survival after further adjustment for age, sex, and abnormal LDH levels (p_coefficient_ < 0.05, LRT *P < 0*.05, 10% FDR, Table S6).

We then analyzed how the log2-FC in plasma levels during treatment was associated with PFS, which could be useful both for prediction of treatment outcome and to indicate the biological effects of these proteins on survival. Adjusted for response to treatment, CCL2 had a log2-FC associated with longer PFS (Figure 2B), replicating the observation in the anti-PD-1 ICB cohort, again showing survival benefit in this group of patents regardless of treatment [13]. Besides CCL2, ADGRG1 was the only other protein that had higher log2-FC associated with longer PFS, adjusted for response to treatment, and was the only protein to remain significant after adjustment for age, sex, and abnormal LDH levels.

In the BRAFi and MEKi subgroup, no clinical variable was associated with PFS, and univariate analyses in HiRIEF LC-MS/MS data showed that higher pre-trm plasma levels of 13 proteins were predictive for PFS, of which 4 proteins were associated with longer PFS: FLNC, MEGF9, ANTXR2, and CP (*P*_coefficient_ < 0.05, LRT *P < 0*.05, no FDR, Figure 2C). The log2-fold change of 38 proteins analyzed with HiRIEF LC-MS/MS was associated with PFS in the BRAFi and MEKi subgroup, of which 19 were associated with longer PFS, including CPB1 (p_coefficient_ < 0.05, LRT *P < 0*.05, no FDR, Figure 2D). Furthermore, 2 proteins with pre-trm levels associated with shorter PFS had increase in their levels during treatment associated with longer PFS – SFTPA and ADGRL4. SNCA was the only protein with log2-fold change associated with PFS in both BRAFi and MEKi treatment (shorter PFS) and anti-PD-1 (longer PFS) [13].

In summary, although limited by the sample size, the results suggest that the proteins associated with PFS might have a role to play in the duration of response to MAPKi treatment and are potential predictive biomarkers.

### mRNA expression in *BRAF^V600E^*-mutated metastatic CM tissue

One challenge when performing a liquid biopsy discovery study in plasma is to pinpoint from where the signal has derived, and if it indeed has a local tumoral origin or rather a systemic immune response origin. Having detected several proteins in plasma that were associated with MAPKi treatment and PFS, we wanted to further explore whether these proteins could have derived from metastatic CM tissue or elsewhere. Due to the lack of an in-depth tissue proteomics dataset with matching survival data, we turned to analyzing TCGA mRNA data as a surrogate for protein expression in CM tissue, to investigate which plasma proteins are traceable to *BRAF^V600E^*-mutated metastatic CM tissue.

**Fig. 3:**
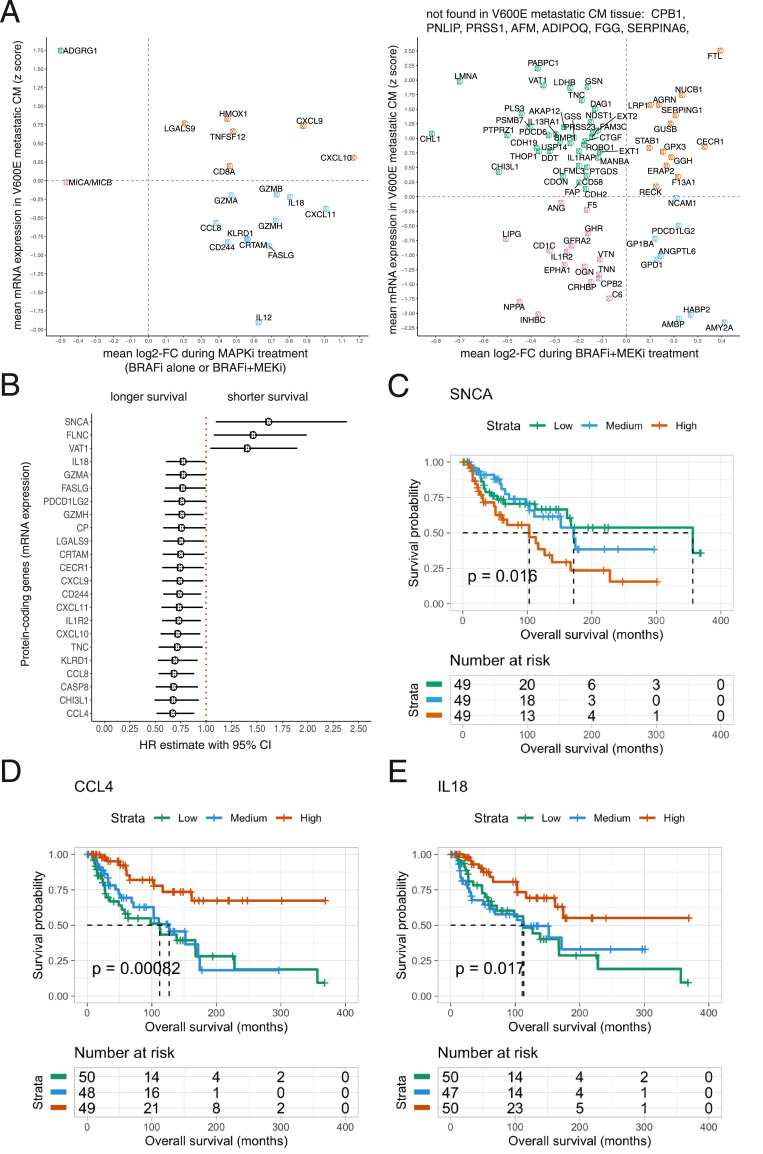
**TCGA analyses.**(A) Proteins with plasma levels’ alterations during MAPKi treatment traceable to *BRAF^V600E−^*mutated metastatic CM tissue at mRNA level; (B) Proteins deregulated in plasma during MAPKi treatment or associated with PFS after MAPKi treatment that have mRNA tissue expression associated with OS in patients with *BRAF^V600E^*-mutated metastatic CM, after adjustment for age and sex (Cox proportional hazards models, *P < 0*.05, 5% FDR); (C) Higher mRNA levels of SNCA in tissue were associated with shorter OS in patients with *BRAF^V600E^*-mutated metastatic CM; (D) Higher mRNA levels of CCL4 in tissue were associated with longer OS in patients with *BRAF^V600E^*-mutated metastatic CM; (E) Higher mRNA levels of IL18 in tissue were associated with longer OS in patients with *BRAF^V600E^*-mutated metastatic CM.

To explore which of the changes in the plasma proteome in the full MAPKi cohort are traceable to tissue, we first calculated the average mRNA expression of a gene in tissue of 154 patients with *BRAF^V600E^*-mutated metastatic CM. The genes were then ranked according to their mean expression after transforming the values to *z* scores, categorizing the gene expression to high (*z* > 0) and low (*z* < 0). All the cytokine proteins altered during treatment in the full MAPKi cohort based on PEA, and 87% of the proteins altered during BRAFi and MEKi treatment were traceable to *BRAF^V600E^* metastatic CM tissue at mRNA level (Figure 3A). Eight proteins that had higher expression in *BRAF^V600E^* metastatic CM tissue and decreased during BRAFi and MEKi treatment belong to the “positive regulation of MAPK cascade” pathway: CHI3L1, DDT, AKAP12, NDST1, CDON, CTGF, CDH2, and ROBO1. However, none of these proteins are exclusively involved in the MAPK pathway.

We found no evidence for expression in tissue of ten proteins altered during BRAFi and MEKi treatment, among them CPB1, PNLIP, PRSS1, SERPINA6, FGG, and F9; all of them increased during treatment and likely not derived from cancer tissue.

### mRNA expression in tissue and association with OS

To further explore the potential tissue origin of the deregulated proteins and proteins associated with PFS detected in the current study, we performed survival analyses at mRNA tissue level in *BRAF^V600E^* metastatic CM from TCGA data with Cox proportional hazards models. The aim of this analysis was to investigate if any of the proteins altered during MAPKi treatment or associated with PFS were also associated with OS at mRNA tissue level in *BRAF^V600E^* metastatic CM. In addition, this allows for differentiation of proteins/genes that have overall an association with OS as well as PFS after MAPKi treatment, thus more likely to be both prognostic and predictive biomarkers, from those only associated with PFS that are more likely predictive only of MAPKi treatment-response duration.

We transformed the protein-coding gene mRNA expression values to *z* scores per patient and adjusted the association between gene expression and OS for age and sex. Among the proteins that were deregulated during MAPKi treatment and/or associated with PFS, the mRNA expression of 23 corresponding genes was associated with OS in patients with *BRAF^V600E^* metastatic CM tissue at 5% FDR (Figure 3B). Of these, alpha synuclein (SNCA) had the strongest association with shorter OS (Figure 3C), whereas chemokine (CC-motif) ligand 4 (CCL4) had the strongest association with longer OS (Figure 3D). Higher levels of IL18 were also associated with longer OS (Figure 3E).

Most of the genes that were associated with longer OS in TCGA data had on average lower expression in *BRAF^V600E^* metastatic CM tissue relative to the remaining genes. It is interesting to observe that most of the corresponding proteins of these lowly expressed genes in *BRAF^V600E^* metastatic CM tissue had an increase in plasma levels during BRAFi or BRAFi and MEKi treatment (Figure 3A). We hypothesize that there is an inverse relation between gene expression in *BRAF^V600E^* metastatic CM tissue and change in corresponding protein plasma levels during MAPKi treatment when it comes to the proteins’ involvement in response to treatment.

## Discussion

This exploratory study aimed at discovering new associations between plasma protein levels, MAPKi treatment, and duration of response to treatment in metastatic *BRAF^V600^*-mutated CM patients.

We recapitulated in plasma some immunomodulating processes in the tumor microenvironment deregulated by *BRAF^V600E^* mutation [21], [22], [23], [24], [25], [26], [27], [28], [29], [30], [31], [32]. The upregulation of CXCL9, CXCL10, and CXCL11, along with the upregulated IL18 indicate upregulation of the IFNγ pathway by MAPKi, whereas the upregulation of GZMA, GZMB and GZMH indicate an upregulation of the granzyme pathways and of cytotoxic T lymphocyte activity. These plasma proteins are likely indicative of generic immune processes stirred by anti-tumor immune response, as observed in anti-PD-1 ICB treatment [13]. It has been established that the presence of *BRAF^V600E^* mutation in CM upregulates the expression of immuno-suppressive molecules such as IL6, IL10, and VEGFA [21,26]. This study shows that higher pre-trm plasma levels of these proteins are also associated with shorter PFS. Although we found that higher pre-trm levels of CCL2 were associated with shorter PFS, our finding that an increase (log2-FC) in CCL2 during treatment is associated with longer PFS are contradicting the findings of Vergani et al, who report that higher CCL2 levels after BRAFi-treatment are associated with a shorter response in CM [33]. However, the analytical approach in our study is different from the one by Vergani et al, because we adjust for pre-trm levels by analyzing the log2-fold change. Still, both results require validation in larger cohorts.

Stratifying the groups by treatment type showed that almost 90% of the signal in the full MAPKi cohort came from the BRAFi subgroup, in which we recapitulate all the proteins that had an increase in plasma levels in the full MAPKi cohort. Still, some of the observations in the BRAFi subgroup are still plausible in the BRAFi and MEKi subgroup. The increase in IL18 seemed consistent in both subgroups. Furthermore, based on TCGA survival analyses of mRNA expression, higher expression of IL18 was associated with survival benefit in metastatic CM patients, suggesting IL18 is a prognostic biomarker.

Analyzing the BRAFi and MEKi subgroup with in-depth proteomics by HiRIEF LC-MS/MS showed alterations in cell adhesion proteins, neutrophil degranulation, and 4 pancreas-associated enzymes. Although most of the proteins deregulated in plasma during treatment were traceable to metastatic CM tissue at mRNA level, it is challenging to prove with certainty that the proteins have derived only from metastatic CM tissue. Although we demonstrate that these genes are expressed in metastatic CM tissue, it is possible that the protein level expression might differ from mRNA expression, as observed in cancer cell lines [34]. It was unexpected to find an increase in pancreas-associated proteins during BRAFi and MEKi treatment. Two of the pancreas-associated proteins, AMY2A and CPB2, were traceable to metastatic CM tissue at mRNA level, but we found no evidence of CPB1 and PNLIP mRNA expression in *BRAF^V600E^* metastatic CM tissue. CPB1 had overall the largest increase during BRAFi and MEKi treatment and was associated with longer PFS. However, CPB1 is abundant in plasma, and considered a biomarker of acute pancreatitis [35]. Its increase in plasma during BRAFi and MEKi treatment could be indicative of subclinical pancreatitis, alike the increase in hepatic enzymes in combined MAPKi and ICB treatment [36,37].

In summary, we detected alterations in plasma levels of several immune-related proteins during MAPKi treatment in patients with *BRAF^V600E^* metastatic CM, which were observable in the BRAFi subgroup as well. Furthermore, we detected signals of proteins involved in cell adhesion, neutrophil degranulation, proteolysis and pancreas-associated enzymes after BRAFi and MEKi treatment of patients with metastatic CM. This explorative study uncovers several proteins that may serve as potential biomarkers of MAPKi treatment response. Our analyses hint that plasma alterations due to BRAFi treatment might be different from those elicited by combined treatment with BRAFi and MEKi, and this should be taken into account in study design and analyses of the plasma proteome.

### Data sharing

Mass spectrometry raw data have been uploaded to the PRIDE repository through the ProteomeXchange, with accession number PXD017201. PEA data are available in Additional File 4 at https://jitc.bmj.com/content/8/1/e000204 [13].

## Author contributions

Conceptualization: Hanna Eriksson, Maria Pernemalm

Data curation: All authors

Formal analysis: Bioinformatics: Haris Babacic.

Additional analysis: All authors.

Funding acquisition: Hanna Eriksson

Investigation: All authors

Methodology: All authors

Project administration: Hanna Eriksson

Resources: Hanna Eriksson, Maria Pernemalm.

Software: Supervision: Hanna Eriksson, Maria Pernemalm.

Validation: All authors

Visualization: All authors.

Roles/Writing - original draft: All authors

Writing - review & editing: All authors

## Appendix Supplementary materials

Image, application 1

Image, application 2
