# Supplementary material for: Plasma proteome alterations by MAPK inhibitors in BRAFV600-mutated metastatic cutaneous melanoma
Source: Neoplasia. 2021 Jul 8;23(8):783–91. doi: 10.1016/j.neo.2021.06.002 (PMC8274243; doi:10.1016/j.neo.2021.06.002)
Supplement: Supplementary file 1 [file mmc1.pdf]

Supplementary File 1

Supplementary methods and figures for study:

Plasma proteome alterations by MAPK inhibitors in *BRAF*^{V600}-mutated metastatic cutaneous melanoma.

Haris Babačić, Hanna Eriksson, Maria Pernemalm. 2021

Supplementary information on Methods

LC-MS/MS analysis

The LC-MS/MS was performed online by Dionex UltiMate™ 3000 RSLCnano System coupled to a Q-Exactive mass spectrometer (Thermo Scientific). The obtained MS/MS files were converted to mzML format by msconvert from the ProteoWizard tool suite [1], and the spectra searched with Galaxy-P tools [2,3], including MSGF+ [4] (v10072) and Percolator [5] (v2.10). For a reference database, we used the human protein subset of ENSEMBL. Quantification on reporter ions in MS2 was performed by the OpenMS IsobaricAnalyzer [6] (v2.0), for both protein and peptide level quantification based on median of PSM ratios, limited to PSMs mapping only to one protein and with an FDR q-value < 0.01. The search settings included enzymatic cleavage of proteins to peptides using trypsin limited to fully tryptic peptides. Carbamidomethylation of cysteine was specified as a fixed modification. The minimum peptide length was specified to be 6 amino acids. Variable modification was oxidation of methionine.

The Cancer Genome Atlas (TCGA) tissue transcriptomics analysis

We downloaded RSEM-normalised, level 3, TCGA HiSeq tumour tissue Illumina mRNA sequencing data and clinical data from 467 patients with CM, from Broad Institute's GDAC Firehouse. Information on *BRAF*^{V600E} and BRAF^{V600K} mutation status and metastatic status were downloaded from Genomic Data Commons (GDC). After removing transcripts with no expression, we transformed the counts to log2-counts per million (log2-CPM) with the *voom* function (*limma* package) [7].

We calculated the *z* score on mean log2-CPM expression per gene transcript, using the mean and the standard deviation (SD) of mean log2-CPM gene expression across patients.

Kaplan-Meier survival analyses. To further confirm the association between gene expression and OS, we categorised the expression levels based on tercile cut-offs: as high (above the upper tercile), medium (above and including the lower tercile), and low (below the lower tercile), and performed survival analysis with Kaplan-Meier curves and a log rank test, at $\alpha = 0.05$.

References:

[1] Kessner D, Chambers M, Burke R, Agus D, Mallick P. ProteoWizard: open source software for rapid proteomics tools development. Bioinformatics 2008;24:2534–6. https://doi.org/10.1093/bioinformatics/btn323.

[2] Boekel J, Chilton JM, Cooke IR, Horvatovich PL, Jagtap PD, Kall L, et al. Multi-omic data analysis using Galaxy. Nat Biotechnol 2015;33:137–9. https://doi.org/10.1038/nbt.3134.

[3] Goecks J, Nekrutenko A, Taylor J. Galaxy: a comprehensive approach for supporting accessible, reproducible, and transparent computational research in the life sciences. Genome Biol 2010;11:R86. https://doi.org/10.1186/gb-2010-11-8-r86.

[4] Kim S, Pevzner PA. MS-GF+ makes progress towards a universal database search tool for proteomics. Nat Commun 2014;5:5277. https://doi.org/10.1038/ncomms6277.

[5] Kall L, Canterbury JD, Weston J, Noble WS, MacCoss MJ. Semi-supervised

learning for peptide identification from shotgun proteomics datasets. Nat Methods 2007;4:923–5. https://doi.org/10.1038/nmeth1113.

[6] Rost HL, Sachsenberg T, Aiche S, Bielow C, Weisser H, Aicheler F, et al. OpenMS: a flexible open-source software platform for mass spectrometry data analysis. Nat Methods 2016;13:741–8. https://doi.org/10.1038/nmeth.3959.

[7] Ritchie ME, Phipson B, Wu D, Hu Y, Law CW, Shi W, et al. limma powers differential expression analyses for RNA-sequencing and microarray studies. Nucleic Acids Res 2015;43:e47. https://doi.org/10.1093/nar/gkv007.

[8] Babačić H, Lehtiö J, Pico De Coaña Y, Pernemalm M, Eriksson H. In-depth plasma proteomics reveals increase in circulating PD-1 during anti-PD-1 immunotherapy in patients with metastatic cutaneous melanoma. J Immunother Cancer 2020. https://doi.org/10.1136/jitc-2019-000204.

Supplementary Figures

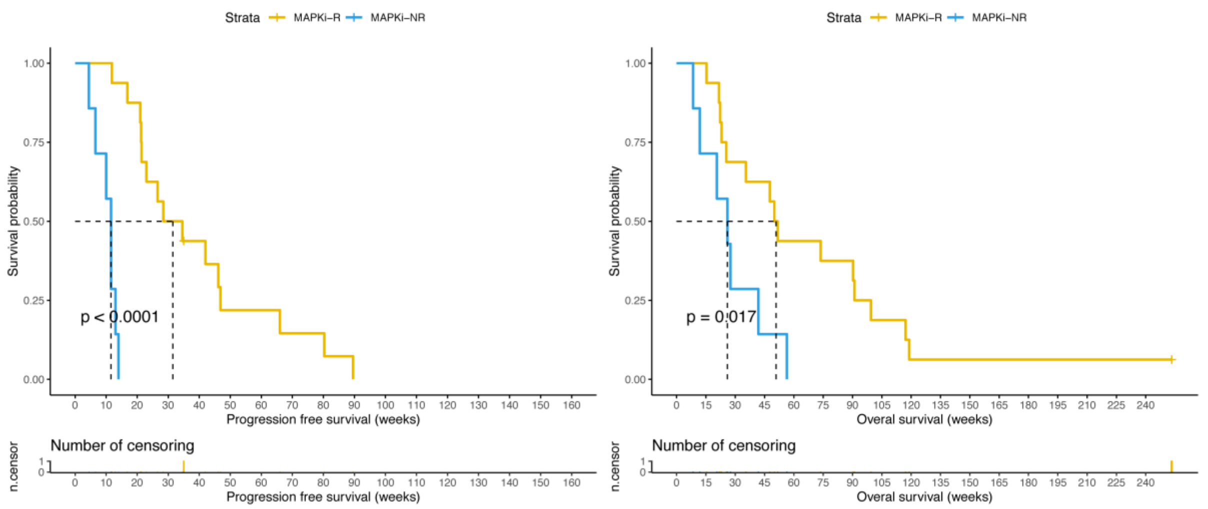

Figure S1. Kaplan-Meier curves on survival in patients receiving MAPKi who have responded to treatment (MAPKis-R) and who have not responded to treatment (MAPKis-NR): progression free survival (lef) and overall survival (right). The dashed lines indicate median survival, p values obtained with a two-sided logrank test.

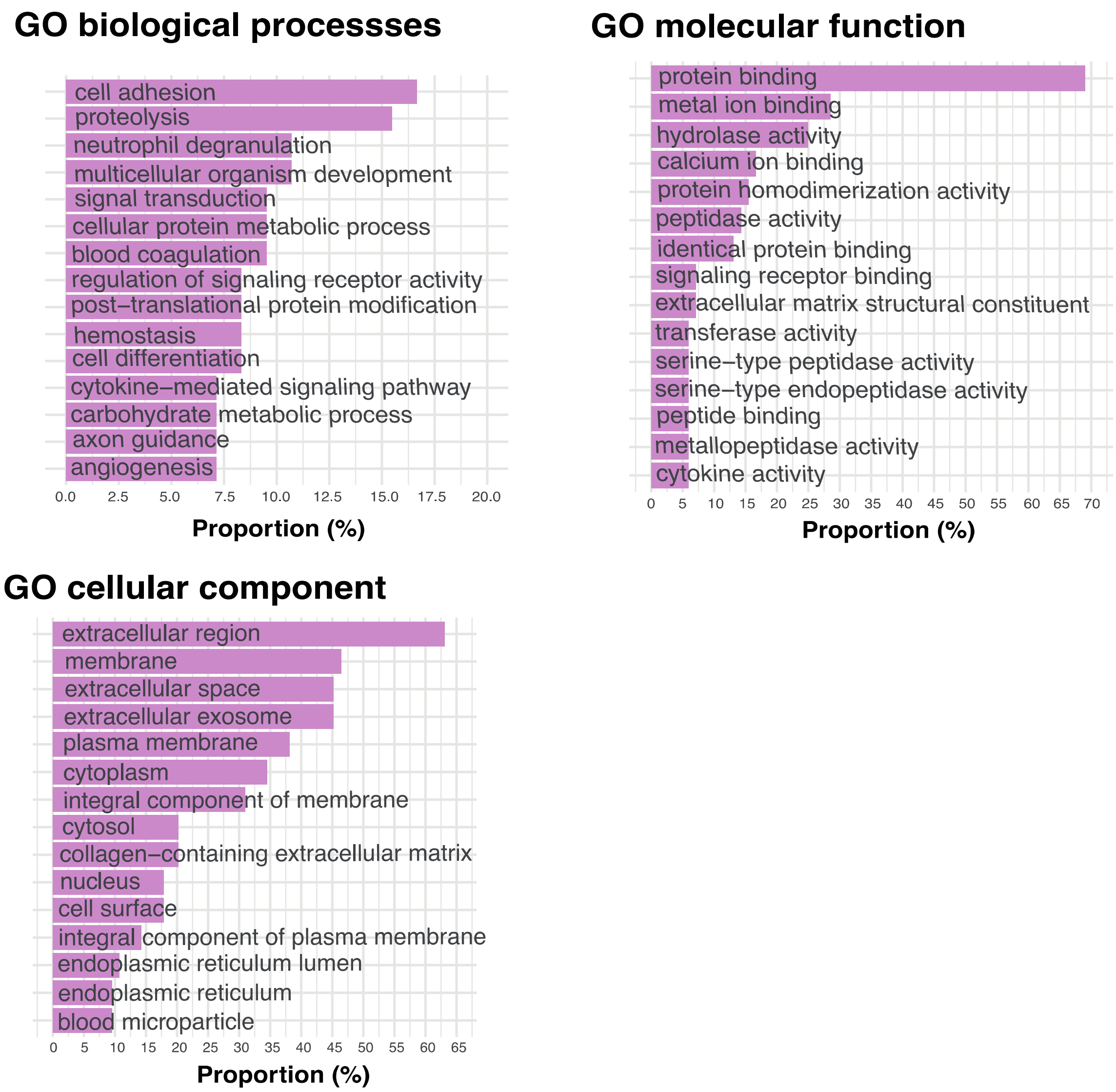

Figure S2. Most frequent gene ontology (GO) terms of the plasma proteins altered during MAPKi-treatment; reused from Babačić *et al.* (2020)[8].
